# Twinned caesium cerium(IV) penta­fluoride

**DOI:** 10.1107/S1600536814003286

**Published:** 2014-02-19

**Authors:** Andrzej Grzechnik, Christopher C. Underwood, Joseph W. Kolis

**Affiliations:** a Institute of Crystallography, RWTH Aachen University, Jägerstr. 17-19, 52066 Aachen, Germany; b Department of Chemistry, Clemson University, Clemson SC 29634, USA

## Abstract

Single-crystals of CsCeF_5_ were synthesized hydro­thermally. The crystal under investigation was twinned by pseudo-merohedry with a twofold rotation around the *c* axis as an additional twinning operation. The crystal structure is built of layers of distorted edge- and corner-sharing CeF_8_ square-anti­prisms. The Cs^+^ cations are located between the layers and exhibit coordination numbers of nine. Upon compression, CsCeF_5_ undergoes an irreversible phase transition at about 1 GPa.

## Related literature   

For background to the applications of complex cerium(IV) and thorium fluorides, see: Friese *et al.* (2011 ▶); Grzechnik *et al.* (2007 ▶, 2008 ▶, 2013*a* ▶,*b* ▶); Rouse & Weller (2009 ▶); Underwood (2013 ▶); Underwood *et al.* (2012 ▶). For related structures, see: Underwood *et al.* (2012 ▶). The ruby luminescence method (Mao *et al.*, 1986 ▶) was used for pressure calibration. According to a recent review of twinning at high pressures (Friese & Grzechnik, 2014 ▶), pseudo-merohedral twinning in general is not significantly affected on compression.

## Experimental   

### 

#### Crystal data  

CsCeF_5_
*M* *_r_* = 368
Monoclinic, 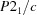

*a* = 8.3125 (5) Å
*b* = 14.2434 (6) Å
*c* = 8.4507 (5) Å
β = 104.566 (5)°
*V* = 968.39 (9) Å^3^
*Z* = 8
Mo *K*α radiation
μ = 16.80 mm^−1^
*T* = 295 K
0.10 × 0.08 × 0.05 mm

#### Data collection  

STOE IPDS 2 diffractometer
Absorption correction: numerical (*X-SHAPE*; Stoe & Cie, 2002 ▶) *T*_min_ = 0.207, *T*_max_ = 0.406
55729 measured reflections
6402 independent reflections
2543 reflections with *I* > 3σ(*I*)
*R*_int_ = 0.136

#### Refinement  

*R*[*F* ^2^ > 2σ(*F* ^2^)] = 0.037
*wR*(*F* ^2^) = 0.038
*S* = 1.04
6402 reflections
129 parameters
Δρ_max_ = 1.85 e Å^−3^
Δρ_min_ = −1.89 e Å^−3^

### 

Data collection: *X-AREA* (Stoe & Cie, 2002 ▶); cell refinement: *X-AREA*; data reduction: *X-AREA*; program(s) used to solve structure: *SIR97* (Altomare *et al.*, 1999 ▶); program(s) used to refine structure: *JANA2006* (Petříček *et al.*, 2006 ▶); molecular graphics: *DIAMOND* (Brandenburg, 2000 ▶); software used to prepare material for publication: *publCIF* (Westrip, 2010 ▶).

## Supplementary Material

Crystal structure: contains datablock(s) global, I. DOI: 10.1107/S1600536814003286/fj2660sup1.cif

Structure factors: contains datablock(s) I. DOI: 10.1107/S1600536814003286/fj2660Isup2.hkl

CCDC reference:

Additional supporting information:  crystallographic information; 3D view; checkCIF report

## Figures and Tables

**Table 1:** Selected bond lengths (Å)

Ce1—F3	2.133 (6)
Ce1—F4	2.304 (5)
Ce1—F5	2.256 (6)
Ce1—F5^i^	2.311 (6)
Ce1—F6	2.320 (5)
Ce1—F7	2.306 (6)
Ce1—F8	2.119 (7)
Ce1—F9	2.487 (6)
Ce1—F9^i^	2.882 (7)
Ce2—F1	2.123 (7)
Ce2—F2	2.128 (7)
Ce2—F4^ii^	2.329 (5)
Ce2—F6	2.395 (6)
Ce2—F6^iii^	3.150 (7)
Ce2—F7^iii^	2.322 (6)
Ce2—F9	2.304 (6)
Ce2—F10	2.242 (5)
Ce2—F10^i^	2.279 (6)
Cs1—F1^iv^	2.928 (8)
Cs1—F2	3.152 (6)
Cs1—F2^v^	2.982 (6)
Cs1—F3^vi^	3.222 (7)
Cs1—F3^iii^	3.229 (7)
Cs1—F5^vi^	3.099 (6)
Cs1—F8	3.095 (6)
Cs1—F8^vii^	3.218 (6)
Cs1—F9	2.982 (5)
Cs2—F1^vi^	2.998 (6)
Cs2—F1^viii^	2.998 (7)
Cs2—F2^ix^	3.042 (8)
Cs2—F3^vi^	3.437 (6)
Cs2—F3^iii^	2.981 (6)
Cs2—F6^vi^	3.125 (6)
Cs2—F7^vii^	3.140 (6)
Cs2—F8	2.980 (7)
Cs2—F10^viii^	3.162 (6)

Symmetry codes: (i) 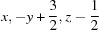; (ii) 
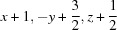
; (iii) 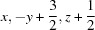; (iv) 
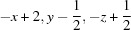
; (v) 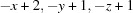; (vi) 
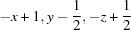
; (vii) 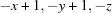; (viii) 
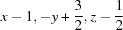
; (ix) 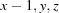.

**Fig. 1.**
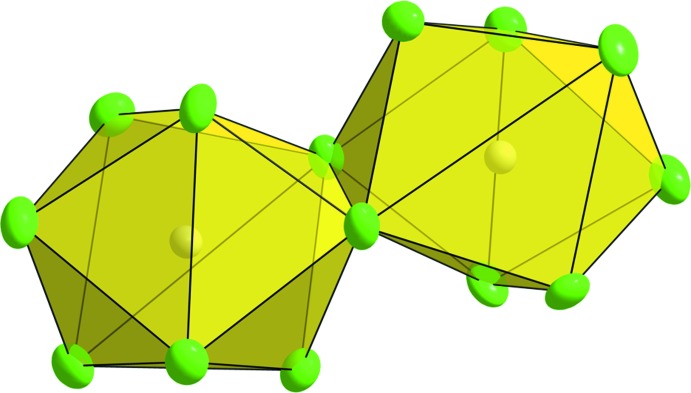
An edge-sharing doublet of distorted square antiprisms CeF8.

**Fig. 2.**
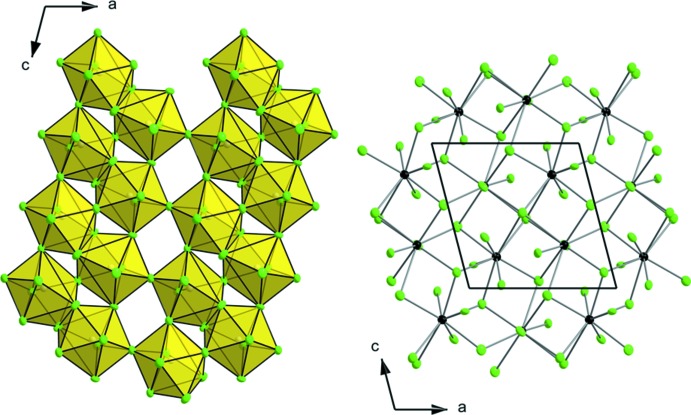
Layers of CeF8 (left) and CsF9 (right) polyhedra viewed along the b axis.

**Fig. 3.**
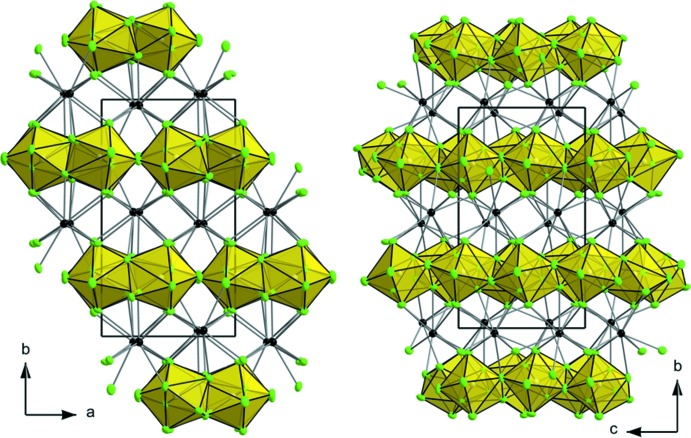
Crystal structure in different projections. The polyhedra around the Ce atoms are drawn.

## supplementary crystallographic information

### 1. Introduction

Most of the work on structural inorganic chemistry of actinide fluorides has been primarily based on the materials that crystallize from molten alkali fluoride salts (Grzechnik *et al.*, 2007; Grzechnik *et al.*, 2008; Friese *et al.*, 2011). Recently, several complex thorium fluorides have been synthesized (Underwood *et al.*, 2012; Grzechnik *et al.*, 2013*a*) and advances in the synthesis of cerium (IV) fluorides have been made (Rouse *et al.*, 2009; Grzechnik *et al.*, 2013*b*). The successful use of fluoride mineralizers in the hydro­thermal crystal growth of alkali thorium fluorides suggests that additional investigations into inorganic cerium (IV) fluorides may be fruitful. The chemistry of monovalent metal cerium(IV) fluorides in hydro­thermal fluids was examined by Underwood (2013) and it was found that it is considerably richer than anti­cipated.

We are inter­ested in the crystal structures and stabilities of cerium (IV) fluorides as they are thought to be isostructural with the actinide fluorides (Grzechnik *et al.*, 2013*b*). In the recent study by Underwood *et al.* (2012), three new polymorphs of CsThF_5_ have been synthesized hydro­thermally. The tetra­gonal phase (phase I, P4/nmm, Z = 2), consisting of sheets of ThF_9_ tricapped trigonal prisms separated by layers of Cs atoms, is a minor product in the hydro­thermal conditions. Two monoclinic polymorphs (phases II and III) are synthesized at various conditions. The phase II (P2_1_/c, Z = 4) is built of chains of corner-sharing ThF_9_ polyhedra, while the phase III (P2_1_/c, Z = 8) is built of sheets of edge- and corner-sharing ThF_9_ polyhedra. The chain structure of the phase II converts into the sheet structure of the phase III at 500°C. In this study, we examine cesium cerium(IV) penta­fluoride, CsCeF_5_, to see whether it is indeed isostructural with CsThF_5_.

### 2. Experimental

A series of crystals mounted on glass pins was tested on a STOE *IPDS* 2 diffractometer and the best one was selected for the structure determination at ambient conditions and high-pressure single-crystal x-ray studies. High-pressure data were collected in the Ahsbahs-type diamond anvil cell at room temperature at 1.2, 3.1, and 5.0 GPa. A 0.250 mm hole was drilled into a stainless steel gasket preindented to a thickness of about 0.120 mm. A 4:1 mixture of methanol and ethanol was used as a pressure medium. The ruby luminescence method (Mao *et al.*, 1986) was used for pressure calibration.

The intensities were indexed and integrated with the software *X-AREA* (Stoe & Cie, 2002). Due to the pseudomerohedral twinning, some reflections in the lower θ range partly overlap. During the integration the orientation matrices of both twin individuals were used simultaneously. The overlap tolerance was set to 100%, so that all overlapped reflections were included. The faces of the crystal were optimized for each individual with the program *X-SHAPE* (Stoe & Cie, 2002) and an absorption correction was applied with the program Jana2006 (Petříček *et al.*, 2006). Figures 1-3 were drawn using the program *DIAMOND* (Brandenburg, 2000).

### 2.1. Synthesis and crystallization

All reagents for the synthesis of CsCeF_5_ were of analytical grade and used as purchased. The compound in this study was prepared hydro­thermally as follows: 0.20 g CeF_4_ (Strem Chemical, 99.9%) was combined with five molar equivalents (0.703 g) of CsF (Alfa Aesar, 99.9%), placed into a Parr Instruments Teflon-lined autoclave and filled with 10.0 mL water. The autoclave was then heated at 250 °C for 24 h and slowly cooled at a rate of 10 °C/h to room temperature. When the reaction was complete, the content of the autoclave was filtered and the product washed with deionized water to yield a colorless material. Powder X-ray diffraction was used to characterize the bulk solid and single crystal X-ray diffraction was used to structurally characterize the new species.

### 2.2. Refinement

Crystal data, data collection and structure refinement details at ambient conditions are summarized in Table 1. The diffraction pattern could be explained assuming a monoclinic lattice with a = 8.3125 (5) Å, b = 14.2434 (6) Å, c = 8.4507 (5) Å, and b = 104.566 (5)° and a two-fold rotation around the c axis as an additional twinning operation. The twinning matrix corresponds to (-1, 0, -0.4948; 0, -1, 0; 0, 0, 1). The twinning could be classified as pseudomerohedral.

The structure was solved with the program SIR97 (Altomare *et al.*, 1999) an it was refined with the program Jana2006 (Petříček *et al.*, 2006). The reflections from both individuals and the twinning operation were taken into account during the refinement process. The refined twin volumes of the two individuals are 0.8830 (7) and 0.1170 (7).

### 3. Results and discussion

All the examined crystals of CsCeF_5_ are pseudomerohedrally twinned and have their lattice parameters close to those for the high-temperature polymorph of CsThF_5_ (phase III) (Underwood *et al.*, 2012). We do not find any material whose single-crystal or powder x-ray intensities could be indexed with the lattices analogous to those in the phases I and II of CsThF_5_. It indicates that CsCeF_5_ does not exhibit any temperature-induced polymorphism within the synthesis conditions.

The analysis of the structural data in Table 2 shows that, unlike the Th atoms in the phase III of CsThF_5_, the Ce atoms are eight-fold coordinated to the fluorine atoms in the distorted square anti­prismatic geometry (Figure 1): the distances Ce1—F and Ce2—F are in the ranges 2.12-2.49 Å and 2.12-2.40 Å, respectively. The average Ce1—F and Ce2—F distances are the same: 2.3 (1) Å. The doublets of edge-sharing CeF_8_ polyhedra share their corners to form sheets perpendicular to the b axis (Figures 2 and 3). The Cs atoms are between the sheets and are coordinated to nine fluorine atoms. The average distances Cs1—F and Cs2—F are identical: 3.1 (1) Å. Altogether, it is clear from our data that, unlike LiCeF_5_ and LiThF_5_ (Grzechnik *et al.*, 2013*b*), CsCeF_5_ and CsThF_5_ are not isostructural at ambient conditions. This suggests that inorganic structural chemistries of actinide and lanthanide (IV) fluorides are not exactly analogous.

Above about 1 GPa, the single-crystal x-ray reflections in CsCeF_5_ lose their intensities, become brodened and smeared, while the crystal itself is cracked into tiny pieces. The reflections can no longer be indexed with any lattice and integrated. These changes in the diffraction pattern are irreversible on decompression to atmospheric conditions. Such a behaviour suggest a pressure-induced first-order phase transition in CsCeF_5_. According to a recent review of twinned structures at high pressures (Friese *et al.*, 2014), pseudomerohedral twinning in general is not significantly affected on compression. The broadening and smearing of the reflections could thus rather be due to the collapse of the layer structure. However, the structure of the pressure-induced form of this material cannot be determined from the current single-crystal x-ray data.

### Crystal data

**Table:**

CsCeF_5_	*F*(000) = 1264
*M**_r_* = 368	*D*_x_ = 5.047 Mg m^−^^3^
Monoclinic, *P*2_1_/*c*	Mo *K*α radiation, λ = 0.71069 Å
Hall symbol: -P 2ybc	Cell parameters from 10057 reflections
*a* = 8.3125 (5) Å	θ = 5.0–32.1°
*b* = 14.2434 (6) Å	µ = 16.80 mm^−^^1^
*c* = 8.4507 (5) Å	*T* = 295 K
β = 104.566 (5)°	Irregular shape, colourless
*V* = 968.39 (9) Å^3^	0.10 × 0.08 × 0.05 mm
*Z* = 8	

### Data collection

**Table:**

STOE IPDS 2 diffractometer	6402 independent reflections
Radiation source: X-ray tube	2543 reflections with *I* > 3σ(*I*)
Plane graphite monochromator	*R*_int_ = 0.136
Detector resolution: 6.67 pixels mm^-1^	θ_max_ = 32.0°, θ_min_ = 4.9°
rotation method scans	*h* = −12→12
Absorption correction: numerical (*X-SHAPE*; Stoe & Cie, 2002)	*k* = −21→21
*T*_min_ = 0.207, *T*_max_ = 0.406	*l* = −12→12
55729 measured reflections	

### Refinement

**Table:**

Refinement on *F*	0 constraints
*R*[*F*^2^ > 2σ(*F*^2^)] = 0.037	Weighting scheme based on measured s.u.'s *w* = 1/(σ^2^(*F*) + 0.0001*F*^2^)
*wR*(*F*^2^) = 0.038	(Δ/σ)_max_ = 0.010
*S* = 1.04	Δρ_max_ = 1.85 e Å^−^^3^
6402 reflections	Δρ_min_ = −1.89 e Å^−^^3^
129 parameters	Extinction correction: B-C type 1 Gaussian isotropic (Becker & Coppens, 1974)
0 restraints	Extinction coefficient: 320 (20)

### Fractional atomic coordinates and isotropic or equivalent isotropic displacement parameters (Å^2^)

**Table:**

	*x*	*y*	*z*	*U*_iso_*/*U*_eq_	
Ce1	0.55769 (5)	0.74670 (6)	0.03297 (5)	0.01796 (12)	
Ce2	0.98279 (5)	0.75627 (6)	0.38963 (5)	0.01810 (12)	
Cs1	0.72055 (8)	0.47560 (6)	0.29747 (8)	0.0279 (2)	
Cs2	0.23935 (7)	0.52409 (6)	0.21659 (8)	0.0285 (2)	
F1	1.0580 (7)	0.8988 (5)	0.3904 (9)	0.031 (2)	
F2	1.0275 (8)	0.6097 (5)	0.4251 (9)	0.030 (2)	
F3	0.5084 (7)	0.8919 (4)	−0.0200 (8)	0.0306 (19)	
F4	0.2716 (6)	0.7548 (6)	−0.0383 (6)	0.0332 (13)	
F5	0.4735 (6)	0.7877 (4)	0.2572 (7)	0.0309 (17)	
F6	0.8051 (7)	0.8239 (4)	0.1490 (7)	0.0286 (17)	
F7	0.7769 (7)	0.6649 (4)	−0.0219 (7)	0.0280 (16)	
F8	0.4839 (7)	0.6055 (5)	0.0505 (8)	0.0311 (19)	
F9	0.7275 (7)	0.6849 (4)	0.2977 (8)	0.0309 (15)	
F10	1.0714 (7)	0.7753 (4)	0.6611 (6)	0.032 (2)	

### Atomic displacement parameters (Å^2^)

**Table:**

	*U*^11^	*U*^22^	*U*^33^	*U*^12^	*U*^13^	*U*^23^
Ce1	0.01799 (17)	0.0192 (2)	0.01669 (18)	−0.0006 (3)	0.00442 (13)	−0.0005 (3)
Ce2	0.01748 (17)	0.0193 (3)	0.01749 (18)	0.0000 (2)	0.00426 (13)	−0.0001 (3)
Cs1	0.0277 (2)	0.0267 (4)	0.0302 (3)	0.0012 (3)	0.0091 (2)	0.0004 (3)
Cs2	0.0279 (3)	0.0285 (4)	0.0285 (3)	−0.0021 (3)	0.0060 (2)	−0.0004 (2)
F1	0.026 (3)	0.031 (4)	0.035 (3)	−0.003 (2)	0.008 (2)	0.001 (3)
F2	0.033 (3)	0.016 (3)	0.041 (4)	0.006 (2)	0.010 (3)	0.001 (3)
F3	0.031 (3)	0.025 (3)	0.037 (3)	0.000 (2)	0.012 (3)	0.005 (3)
F4	0.0222 (16)	0.049 (3)	0.0287 (17)	0.006 (4)	0.0073 (15)	0.004 (5)
F5	0.022 (2)	0.040 (3)	0.031 (3)	0.007 (2)	0.008 (2)	0.005 (3)
F6	0.025 (2)	0.025 (3)	0.032 (3)	−0.003 (2)	−0.001 (2)	0.004 (2)
F7	0.022 (2)	0.029 (3)	0.034 (3)	0.003 (2)	0.010 (2)	0.003 (2)
F8	0.031 (3)	0.025 (3)	0.036 (3)	−0.004 (2)	0.006 (2)	0.005 (3)
F9	0.028 (2)	0.022 (2)	0.036 (3)	−0.002 (2)	−0.004 (2)	0.008 (3)
F10	0.030 (2)	0.050 (5)	0.016 (2)	0.001 (2)	0.0065 (18)	0.005 (2)

### Geometric parameters (Å, º)

**Table:**

Ce1—F3	2.133 (6)	F1—F8^ii^	3.458 (8)
Ce1—F4	2.304 (5)	F1—F10	2.865 (9)
Ce1—F5	2.256 (6)	F1—F10^i^	3.166 (10)
Ce1—F5^i^	2.311 (6)	F2—F2^v^	3.445 (10)
Ce1—F6	2.320 (5)	F2—F4^ii^	2.761 (10)
Ce1—F7	2.306 (6)	F2—F6^iii^	3.107 (10)
Ce1—F8	2.119 (7)	F2—F9	2.679 (8)
Ce1—F9	2.487 (6)	F2—F10	3.051 (9)
Ce1—F9^i^	2.882 (7)	F2—F10^i^	2.864 (9)
Ce2—F1	2.123 (7)	F3—F3^x^	3.106 (9)
Ce2—F2	2.128 (7)	F3—F4	2.749 (10)
Ce2—F4^ii^	2.329 (5)	F3—F5	2.848 (9)
Ce2—F6	2.395 (6)	F3—F5^i^	3.146 (9)
Ce2—F6^iii^	3.150 (7)	F3—F6	2.700 (8)
Ce2—F7^iii^	2.322 (6)	F3—F9^i^	2.881 (10)
Ce2—F9	2.304 (6)	F4—F5	2.676 (7)
Ce2—F10	2.242 (5)	F4—F5^i^	2.762 (9)
Ce2—F10^i^	2.279 (6)	F4—F8	2.745 (10)
Cs1—F1^iv^	2.928 (8)	F4—F10^xi^	2.680 (6)
Cs1—F2	3.152 (6)	F4—F10^viii^	2.684 (8)
Cs1—F2^v^	2.982 (6)	F5—F6	3.157 (9)
Cs1—F3^vi^	3.222 (7)	F5—F7^iii^	2.817 (7)
Cs1—F3^iii^	3.229 (7)	F5—F8	3.142 (9)
Cs1—F5^vi^	3.099 (6)	F5—F8^iii^	2.891 (9)
Cs1—F8	3.095 (6)	F5—F9	2.521 (8)
Cs1—F8^vii^	3.218 (6)	F5—F10^viii^	3.357 (8)
Cs1—F9	2.982 (5)	F6—F7	2.664 (8)
Cs2—F1^vi^	2.998 (6)	F6—F7^iii^	2.853 (9)
Cs2—F1^viii^	2.998 (7)	F6—F9	2.514 (9)
Cs2—F2^ix^	3.042 (8)	F6—F9^i^	2.878 (9)
Cs2—F3^vi^	3.437 (6)	F6—F10^i^	2.607 (8)
Cs2—F3^iii^	2.981 (6)	F7—F8	2.788 (9)
Cs2—F6^vi^	3.125 (6)	F7—F9	2.847 (9)
Cs2—F7^vii^	3.140 (6)	F7—F9^i^	2.598 (8)
Cs2—F8	2.980 (7)	F7—F10^i^	2.684 (7)
Cs2—F10^viii^	3.162 (6)	F8—F8^vii^	3.153 (9)
F1—F4^ii^	2.786 (10)	F8—F9	2.760 (8)
F1—F6	2.750 (8)	F9—F10^i^	3.390 (9)
F1—F7^iii^	2.776 (9)		
			
F3—Ce1—F4	76.4 (3)	F3^iii^—F5—F6	110.5 (2)
F3—Ce1—F5	80.9 (2)	F3^iii^—F5—F7^iii^	82.3 (2)
F3—Ce1—F5^i^	90.0 (2)	F3^iii^—F5—F8	69.5 (2)
F3—Ce1—F6	74.5 (2)	F3^iii^—F5—F8^iii^	86.3 (2)
F3—Ce1—F7	124.6 (2)	F3^iii^—F5—F9	59.9 (2)
F3—Ce1—F8	153.0 (2)	F3^iii^—F5—F10^viii^	82.2 (2)
F3—Ce1—F9	124.6 (2)	F4—F5—F4^iii^	102.0 (2)
F3—Ce1—F9^i^	68.3 (2)	F4—F5—F6	98.4 (2)
F4—Ce1—F5	71.86 (18)	F4—F5—F7^iii^	155.1 (3)
F4—Ce1—F5^i^	73.51 (19)	F4—F5—F8	55.6 (2)
F4—Ce1—F6	146.4 (3)	F4—F5—F8^iii^	140.2 (3)
F4—Ce1—F7	142.0 (2)	F4—F5—F9	109.4 (3)
F4—Ce1—F8	76.6 (3)	F4—F5—F10^viii^	51.32 (17)
F4—Ce1—F9	124.6 (2)	F4^iii^—F5—F6	158.3 (2)
F4—Ce1—F9^i^	117.07 (18)	F4^iii^—F5—F7^iii^	102.8 (2)
F5—Ce1—F5^i^	145.35 (18)	F4^iii^—F5—F8	105.8 (3)
F5—Ce1—F6	87.2 (2)	F4^iii^—F5—F8^iii^	58.0 (2)
F5—Ce1—F7	136.69 (19)	F4^iii^—F5—F9	114.1 (3)
F5—Ce1—F8	91.8 (2)	F4^iii^—F5—F10^viii^	50.83 (16)
F5—Ce1—F9	64.0 (2)	F6—F5—F7^iii^	56.7 (2)
F5—Ce1—F9^i^	143.25 (19)	F6—F5—F8	80.0 (2)
F5^i^—Ce1—F6	122.6 (2)	F6—F5—F8^iii^	108.8 (2)
F5^i^—Ce1—F7	75.19 (19)	F6—F5—F9	51.1 (2)
F5^i^—Ce1—F8	81.4 (2)	F6—F5—F10^viii^	149.6 (2)
F5^i^—Ce1—F9	142.4 (2)	F7^iii^—F5—F8	114.9 (2)
F5^i^—Ce1—F9^i^	56.81 (18)	F7^iii^—F5—F8^iii^	58.5 (2)
F6—Ce1—F7	70.3 (2)	F7^iii^—F5—F9	57.9 (2)
F6—Ce1—F8	131.4 (2)	F7^iii^—F5—F10^viii^	153.6 (3)
F6—Ce1—F9	62.9 (2)	F8—F5—F8^iii^	155.8 (3)
F6—Ce1—F9^i^	66.2 (2)	F8—F5—F9	57.1 (2)
F7—Ce1—F8	78.0 (2)	F8—F5—F10^viii^	79.02 (19)
F7—Ce1—F9	72.8 (2)	F8^iii^—F5—F9	110.2 (3)
F7—Ce1—F9^i^	58.87 (18)	F8^iii^—F5—F10^viii^	99.3 (2)
F8—Ce1—F9	73.1 (2)	F9—F5—F10^viii^	128.8 (3)
F8—Ce1—F9^i^	124.7 (2)	Ce1—F6—Ce2	117.4 (3)
F9—Ce1—F9^i^	118.33 (19)	Ce1—F6—Ce2^i^	93.16 (19)
F1—Ce2—F2	153.7 (2)	Ce1—F6—Cs2^xiii^	114.4 (2)
F1—Ce2—F4^ii^	77.3 (3)	Ce1—F6—F1	158.2 (3)
F1—Ce2—F6	74.7 (2)	Ce1—F6—F2^i^	119.1 (3)
F1—Ce2—F6^iii^	122.3 (2)	Ce1—F6—F3	49.59 (17)
F1—Ce2—F7^iii^	77.1 (2)	Ce1—F6—F5	45.54 (15)
F1—Ce2—F9	131.9 (2)	Ce1—F6—F7	54.58 (17)
F1—Ce2—F10	82.0 (3)	Ce1—F6—F7^iii^	99.0 (3)
F1—Ce2—F10^i^	91.9 (3)	Ce1—F6—F9	61.76 (19)
F2—Ce2—F4^ii^	76.4 (3)	Ce1—F6—F9^i^	66.33 (18)
F2—Ce2—F6	124.2 (2)	Ce1—F6—F10^i^	114.4 (3)
F2—Ce2—F6^iii^	69.0 (2)	Ce2—F6—Ce2^i^	98.51 (19)
F2—Ce2—F7^iii^	123.1 (3)	Ce2—F6—Cs2^xiii^	99.35 (19)
F2—Ce2—F9	74.2 (2)	Ce2—F6—F1	48.14 (18)
F2—Ce2—F10	88.5 (2)	Ce2—F6—F2^i^	107.8 (2)
F2—Ce2—F10^i^	81.0 (3)	Ce2—F6—F3	151.6 (3)
F4^ii^—Ce2—F6	128.6 (2)	Ce2—F6—F5	94.9 (2)
F4^ii^—Ce2—F6^iii^	115.26 (18)	Ce2—F6—F7	94.0 (2)
F4^ii^—Ce2—F7^iii^	138.0 (2)	Ce2—F6—F7^iii^	51.62 (16)
F4^ii^—Ce2—F9	149.3 (3)	Ce2—F6—F9	55.94 (18)
F4^ii^—Ce2—F10	71.9 (2)	Ce2—F6—F9^i^	142.1 (3)
F4^ii^—Ce2—F10^i^	71.13 (18)	Ce2—F6—F10^i^	54.00 (16)
F6—Ce2—F6^iii^	116.07 (18)	Ce2^i^—F6—Cs2^xiii^	134.6 (2)
F6—Ce2—F7^iii^	74.4 (2)	Ce2^i^—F6—F1	104.6 (2)
F6—Ce2—F9	64.7 (2)	Ce2^i^—F6—F2^i^	39.77 (15)
F6—Ce2—F10	142.5 (2)	Ce2^i^—F6—F3	106.7 (2)
F6—Ce2—F10^i^	67.8 (2)	Ce2^i^—F6—F5	137.6 (2)
F6^iii^—Ce2—F7^iii^	55.83 (18)	Ce2^i^—F6—F7	46.15 (17)
F6^iii^—Ce2—F9	61.40 (19)	Ce2^i^—F6—F7^iii^	150.0 (2)
F6^iii^—Ce2—F10	54.76 (18)	Ce2^i^—F6—F9	106.7 (2)
F6^iii^—Ce2—F10^i^	145.75 (19)	Ce2^i^—F6—F9^i^	44.67 (15)
F7^iii^—Ce2—F9	68.3 (2)	Ce2^i^—F6—F10^i^	44.62 (15)
F7^iii^—Ce2—F10	72.0 (2)	Cs2^xiii^—F6—F1	60.97 (19)
F7^iii^—Ce2—F10^i^	142.19 (19)	Cs2^xiii^—F6—F2^i^	94.9 (2)
F9—Ce2—F10	115.9 (2)	Cs2^xiii^—F6—F3	71.90 (19)
F9—Ce2—F10^i^	95.4 (2)	Cs2^xiii^—F6—F5	81.50 (19)
F10—Ce2—F10^i^	142.9 (2)	Cs2^xiii^—F6—F7	166.0 (2)
F1^iv^—Cs1—F2	81.20 (19)	Cs2^xiii^—F6—F7^iii^	63.18 (17)
F1^iv^—Cs1—F2^v^	82.0 (2)	Cs2^xiii^—F6—F9	117.9 (3)
F1^iv^—Cs1—F3^vi^	136.22 (19)	Cs2^xiii^—F6—F9^i^	113.5 (2)
F1^iv^—Cs1—F3^iii^	166.16 (19)	Cs2^xiii^—F6—F10^i^	131.1 (2)
F1^iv^—Cs1—F5^vi^	88.34 (18)	F1—F6—F2^i^	82.7 (2)
F1^iv^—Cs1—F8	103.73 (19)	F1—F6—F3	132.8 (3)
F1^iv^—Cs1—F8^vii^	68.29 (17)	F1—F6—F5	114.1 (3)
F1^iv^—Cs1—F9	111.1 (2)	F1—F6—F7	132.7 (3)
F2—Cs1—F2^v^	68.27 (17)	F1—F6—F7^iii^	59.4 (2)
F2—Cs1—F3^vi^	124.79 (18)	F1—F6—F9	100.3 (3)
F2—Cs1—F3^iii^	88.55 (17)	F1—F6—F9^i^	135.5 (3)
F2—Cs1—F5^vi^	156.99 (15)	F1—F6—F10^i^	72.4 (2)
F2—Cs1—F8	101.01 (16)	F2^i^—F6—F3	100.0 (3)
F2—Cs1—F8^vii^	136.56 (19)	F2^i^—F6—F5	157.3 (2)
F2—Cs1—F9	51.69 (16)	F2^i^—F6—F7	85.0 (3)
F2^v^—Cs1—F3^vi^	77.96 (18)	F2^i^—F6—F7^iii^	141.6 (3)
F2^v^—Cs1—F3^iii^	102.87 (18)	F2^i^—F6—F9	144.2 (3)
F2^v^—Cs1—F5^vi^	90.09 (16)	F2^i^—F6—F9^i^	53.0 (2)
F2^v^—Cs1—F8	167.23 (17)	F2^i^—F6—F10^i^	63.8 (2)
F2^v^—Cs1—F8^vii^	132.64 (17)	F3—F6—F5	57.6 (2)
F2^v^—Cs1—F9	113.41 (16)	F3—F6—F7	94.2 (2)
F3^vi^—Cs1—F3^iii^	57.57 (16)	F3—F6—F7^iii^	102.0 (3)
F3^vi^—Cs1—F5^vi^	53.52 (16)	F3—F6—F9	103.4 (3)
F3^vi^—Cs1—F8	104.26 (16)	F3—F6—F9^i^	62.1 (2)
F3^vi^—Cs1—F8^vii^	98.44 (16)	F3—F6—F10^i^	150.3 (3)
F3^vi^—Cs1—F9	112.58 (18)	F5—F6—F7	93.1 (2)
F3^iii^—Cs1—F5^vi^	104.46 (16)	F5—F6—F7^iii^	55.62 (19)
F3^iii^—Cs1—F8	69.01 (17)	F5—F6—F9	51.3 (2)
F3^iii^—Cs1—F8^vii^	114.83 (15)	F5—F6—F9^i^	107.8 (2)
F3^iii^—Cs1—F9	55.10 (18)	F5—F6—F10^i^	134.0 (3)
F5^vi^—Cs1—F8	101.36 (15)	F7—F6—F7^iii^	123.9 (3)
F5^vi^—Cs1—F8^vii^	54.44 (16)	F7—F6—F9	66.6 (2)
F5^vi^—Cs1—F9	150.79 (15)	F7—F6—F9^i^	55.8 (2)
F8—Cs1—F8^vii^	59.89 (16)	F7—F6—F10^i^	61.2 (2)
F8—Cs1—F9	53.97 (16)	F7^iii^—F6—F9	57.5 (2)
F8^vii^—Cs1—F9	111.41 (16)	F7^iii^—F6—F9^i^	162.9 (3)
F1^vi^—Cs2—F1^viii^	75.84 (19)	F7^iii^—F6—F10^i^	105.6 (2)
F1^vi^—Cs2—F2^ix^	81.94 (19)	F9—F6—F9^i^	117.5 (3)
F1^vi^—Cs2—F3^vi^	101.58 (17)	F9—F6—F10^i^	82.9 (3)
F1^vi^—Cs2—F3^iii^	150.6 (2)	F9^i^—F6—F10^i^	89.1 (3)
F1^vi^—Cs2—F6^vi^	53.32 (16)	Ce1—F7—Ce2^i^	120.3 (2)
F1^vi^—Cs2—F7^vii^	53.71 (18)	Ce1—F7—Cs2^vii^	127.3 (2)
F1^vi^—Cs2—F8	134.27 (19)	Ce1—F7—F1^i^	168.5 (3)
F1^vi^—Cs2—F10^viii^	101.15 (17)	Ce1—F7—F5^i^	52.49 (17)
F1^viii^—Cs2—F2^ix^	98.94 (19)	Ce1—F7—F6	55.10 (18)
F1^viii^—Cs2—F3^vi^	163.34 (19)	Ce1—F7—F6^i^	115.5 (2)
F1^viii^—Cs2—F3^iii^	130.44 (18)	Ce1—F7—F8	48.02 (18)
F1^viii^—Cs2—F6^vi^	126.19 (17)	Ce1—F7—F9	56.55 (18)
F1^viii^—Cs2—F7^vii^	83.52 (17)	Ce1—F7—F9^i^	71.7 (2)
F1^viii^—Cs2—F8	70.68 (17)	Ce1—F7—F10^i^	112.1 (3)
F1^viii^—Cs2—F10^viii^	55.35 (17)	Ce2^i^—F7—Cs2^vii^	100.6 (2)
F2^ix^—Cs2—F3^vi^	96.96 (17)	Ce2^i^—F7—F1^i^	48.22 (19)
F2^ix^—Cs2—F3^iii^	80.87 (18)	Ce2^i^—F7—F5^i^	106.3 (2)
F2^ix^—Cs2—F6^vi^	90.96 (18)	Ce2^i^—F7—F6	78.0 (2)
F2^ix^—Cs2—F7^vii^	133.81 (17)	Ce2^i^—F7—F6^i^	53.95 (18)
F2^ix^—Cs2—F8	132.82 (18)	Ce2^i^—F7—F8	166.6 (3)
F2^ix^—Cs2—F10^viii^	54.94 (17)	Ce2^i^—F7—F9	123.4 (2)
F3^vi^—Cs2—F3^iii^	57.36 (16)	Ce2^i^—F7—F9^i^	55.52 (19)
F3^vi^—Cs2—F6^vi^	48.29 (14)	Ce2^i^—F7—F10^i^	52.62 (17)
F3^vi^—Cs2—F7^vii^	81.88 (15)	Cs2^vii^—F7—F1^i^	60.53 (18)
F3^vi^—Cs2—F8	101.76 (16)	Cs2^vii^—F7—F5^i^	86.83 (19)
F3^vi^—Cs2—F10^viii^	140.30 (14)	Cs2^vii^—F7—F6	177.4 (2)
F3^iii^—Cs2—F6^vi^	103.30 (16)	Cs2^vii^—F7—F6^i^	62.65 (17)
F3^iii^—Cs2—F7^vii^	131.37 (16)	Cs2^vii^—F7—F8	86.0 (2)
F3^iii^—Cs2—F8	73.91 (18)	Cs2^vii^—F7—F9	125.8 (2)
F3^iii^—Cs2—F10^viii^	88.18 (15)	Cs2^vii^—F7—F9^i^	114.8 (2)
F6^vi^—Cs2—F7^vii^	54.18 (16)	Cs2^vii^—F7—F10^i^	119.1 (2)
F6^vi^—Cs2—F8	133.07 (17)	F1^i^—F7—F5^i^	125.1 (3)
F6^vi^—Cs2—F10^viii^	142.19 (16)	F1^i^—F7—F6	117.2 (3)
F7^vii^—Cs2—F8	91.80 (17)	F1^i^—F7—F6^i^	58.5 (2)
F7^vii^—Cs2—F10^viii^	137.52 (13)	F1^i^—F7—F8	143.1 (3)
F8—Cs2—F10^viii^	84.64 (17)	F1^i^—F7—F9	128.2 (3)
Ce2—F1—Cs1^xii^	125.5 (3)	F1^i^—F7—F9^i^	97.6 (3)
Ce2—F1—Cs2^xiii^	110.4 (2)	F1^i^—F7—F10^i^	63.3 (2)
Ce2—F1—Cs2^ii^	115.6 (3)	F5^i^—F7—F6	95.6 (2)
Ce2—F1—F4^ii^	54.7 (2)	F5^i^—F7—F6^i^	67.7 (2)
Ce2—F1—F6	57.14 (19)	F5^i^—F7—F8	62.1 (2)
Ce2—F1—F7^iii^	54.6 (2)	F5^i^—F7—F9	106.6 (3)
Ce2—F1—F8^ii^	104.8 (3)	F5^i^—F7—F9^i^	55.3 (2)
Ce2—F1—F10	50.81 (19)	F5^i^—F7—F10^i^	147.2 (3)
Ce2—F1—F10^i^	45.99 (18)	F6—F7—F6^i^	117.6 (3)
Cs1^xii^—F1—Cs2^xiii^	101.4 (2)	F6—F7—F8	95.8 (3)
Cs1^xii^—F1—Cs2^ii^	97.03 (18)	F6—F7—F9	54.2 (2)
Cs1^xii^—F1—F4^ii^	87.6 (2)	F6—F7—F9^i^	66.3 (2)
Cs1^xii^—F1—F6	102.2 (3)	F6—F7—F10^i^	58.4 (2)
Cs1^xii^—F1—F7^iii^	162.4 (3)	F6^i^—F7—F8	121.4 (2)
Cs1^xii^—F1—F8^ii^	59.84 (16)	F6^i^—F7—F9	170.4 (3)
Cs1^xii^—F1—F10	139.3 (3)	F6^i^—F7—F9^i^	54.7 (2)
Cs1^xii^—F1—F10^i^	80.6 (2)	F6^i^—F7—F10^i^	104.9 (3)
Cs2^xiii^—F1—Cs2^ii^	104.2 (2)	F8—F7—F9	58.6 (2)
Cs2^xiii^—F1—F4^ii^	164.8 (3)	F8—F7—F9^i^	111.2 (3)
Cs2^xiii^—F1—F6	65.71 (18)	F8—F7—F10^i^	133.7 (3)
Cs2^xiii^—F1—F7^iii^	65.76 (18)	F9—F7—F9^i^	115.8 (3)
Cs2^xiii^—F1—F8^ii^	144.5 (3)	F9—F7—F10^i^	75.5 (2)
Cs2^xiii^—F1—F10	118.0 (3)	F9^i^—F7—F10^i^	93.7 (2)
Cs2^xiii^—F1—F10^i^	115.8 (2)	Ce1—F8—Cs1	117.9 (2)
Cs2^ii^—F1—F4^ii^	86.7 (2)	Ce1—F8—Cs1^vii^	111.9 (2)
Cs2^ii^—F1—F6	159.7 (3)	Ce1—F8—Cs2	130.2 (3)
Cs2^ii^—F1—F7^iii^	97.8 (3)	Ce1—F8—F1^viii^	106.0 (2)
Cs2^ii^—F1—F8^ii^	54.42 (15)	Ce1—F8—F4	54.7 (2)
Cs2^ii^—F1—F10	65.24 (19)	Ce1—F8—F5	45.86 (17)
Cs2^ii^—F1—F10^i^	139.7 (2)	Ce1—F8—F5^i^	52.21 (18)
F4^ii^—F1—F6	100.5 (3)	Ce1—F8—F7	54.00 (19)
F4^ii^—F1—F7^iii^	102.7 (3)	Ce1—F8—F8^vii^	147.1 (3)
F4^ii^—F1—F8^ii^	50.8 (2)	Ce1—F8—F9	59.58 (19)
F4^ii^—F1—F10	56.7 (2)	Cs1—F8—Cs1^vii^	120.1 (2)
F4^ii^—F1—F10^i^	53.06 (19)	Cs1—F8—Cs2	80.98 (17)
F6—F1—F7^iii^	62.2 (2)	Cs1—F8—F1^viii^	131.9 (2)
F6—F1—F8^ii^	143.1 (3)	Cs1—F8—F4	154.6 (3)
F6—F1—F10	102.9 (3)	Cs1—F8—F5	102.8 (2)
F6—F1—F10^i^	51.7 (2)	Cs1—F8—F5^i^	140.3 (3)
F7^iii^—F1—F8^ii^	137.5 (3)	Cs1—F8—F7	83.54 (19)
F7^iii^—F1—F10	56.8 (2)	Cs1—F8—F8^vii^	61.99 (16)
F7^iii^—F1—F10^i^	94.0 (3)	Cs1—F8—F9	60.92 (16)
F8^ii^—F1—F10	81.2 (2)	Cs1^vii^—F8—Cs2	91.41 (16)
F8^ii^—F1—F10^i^	92.0 (2)	Cs1^vii^—F8—F1^viii^	51.88 (15)
F10—F1—F10^i^	90.4 (3)	Cs1^vii^—F8—F4	82.68 (19)
Ce2—F2—Cs1	116.7 (2)	Cs1^vii^—F8—F5	136.2 (2)
Ce2—F2—Cs1^v^	125.0 (3)	Cs1^vii^—F8—F5^i^	60.67 (16)
Ce2—F2—Cs2^xiv^	114.7 (3)	Cs1^vii^—F8—F7	101.4 (2)
Ce2—F2—F2^v^	155.1 (3)	Cs1^vii^—F8—F8^vii^	58.11 (15)
Ce2—F2—F4^ii^	55.1 (2)	Cs1^vii^—F8—F9	163.2 (3)
Ce2—F2—F6^iii^	71.2 (2)	Cs2—F8—F1^viii^	54.90 (16)
Ce2—F2—F9	55.89 (18)	Cs2—F8—F4	87.8 (2)
Ce2—F2—F10	47.28 (17)	Cs2—F8—F5	86.7 (2)
Ce2—F2—F10^i^	51.8 (2)	Cs2—F8—F5^i^	137.0 (2)
Cs1—F2—Cs1^v^	111.7 (2)	Cs2—F8—F7	163.5 (3)
Cs1—F2—Cs2^xiv^	95.48 (19)	Cs2—F8—F8^vii^	82.6 (2)
Cs1—F2—F2^v^	53.51 (14)	Cs2—F8—F9	105.1 (3)
Cs1—F2—F4^ii^	166.1 (3)	F1^viii^—F8—F4	51.8 (2)
Cs1—F2—F6^iii^	80.7 (2)	F1^viii^—F8—F5	93.6 (2)
Cs1—F2—F9	60.88 (17)	F1^viii^—F8—F5^i^	82.3 (2)
Cs1—F2—F10	130.2 (3)	F1^viii^—F8—F7	141.6 (3)
Cs1—F2—F10^i^	109.3 (2)	F1^viii^—F8—F8^vii^	91.5 (2)
Cs1^v^—F2—Cs2^xiv^	84.12 (17)	F1^viii^—F8—F9	141.9 (3)
Cs1^v^—F2—F2^v^	58.21 (15)	F4—F8—F5	53.57 (19)
Cs1^v^—F2—F4^ii^	81.1 (2)	F4—F8—F5^i^	58.6 (2)
Cs1^v^—F2—F6^iii^	93.0 (2)	F4—F8—F7	104.0 (3)
Cs1^v^—F2—F9	151.4 (3)	F4—F8—F8^vii^	139.2 (3)
Cs1^v^—F2—F10	81.74 (19)	F4—F8—F9	100.8 (3)
Cs1^v^—F2—F10^i^	130.1 (3)	F5—F8—F5^i^	92.5 (2)
Cs2^xiv^—F2—F2^v^	89.9 (2)	F5—F8—F7	91.1 (2)
Cs2^xiv^—F2—F4^ii^	80.1 (2)	F5—F8—F8^vii^	162.5 (3)
Cs2^xiv^—F2—F6^iii^	174.1 (3)	F5—F8—F9	50.06 (18)
Cs2^xiv^—F2—F9	122.9 (3)	F5^i^—F8—F7	59.4 (2)
Cs2^xiv^—F2—F10	134.2 (3)	F5^i^—F8—F8^vii^	104.8 (3)
Cs2^xiv^—F2—F10^i^	64.7 (2)	F5^i^—F8—F9	106.9 (3)
F2^v^—F2—F4^ii^	139.0 (3)	F7—F8—F8^vii^	95.3 (3)
F2^v^—F2—F6^iii^	84.2 (2)	F7—F8—F9	61.7 (2)
F2^v^—F2—F9	108.3 (3)	F8^vii^—F8—F9	120.0 (2)
F2^v^—F2—F10	117.8 (3)	Ce1—F9—Ce1^iii^	103.6 (2)
F2^v^—F2—F10^i^	149.3 (3)	Ce1—F9—Ce2	114.4 (3)
F4^ii^—F2—F6^iii^	104.6 (3)	Ce1—F9—Cs1	110.27 (19)
F4^ii^—F2—F9	110.4 (3)	Ce1—F9—F2	139.8 (3)
F4^ii^—F2—F10	54.7 (2)	Ce1—F9—F3^iii^	108.9 (2)
F4^ii^—F2—F10^i^	56.9 (2)	Ce1—F9—F5	53.54 (18)
F6^iii^—F2—F9	59.1 (2)	Ce1—F9—F6	55.29 (17)
F6^iii^—F2—F10	50.09 (19)	Ce1—F9—F6^iii^	150.8 (3)
F6^iii^—F2—F10^i^	120.8 (3)	Ce1—F9—F7	50.68 (16)
F9—F2—F10	84.2 (2)	Ce1—F9—F7^iii^	101.9 (2)
F9—F2—F10^i^	75.3 (2)	Ce1—F9—F8	47.29 (17)
F10—F2—F10^i^	92.8 (2)	Ce1—F9—F10^i^	88.3 (2)
Ce1—F3—Cs1^xiii^	110.3 (3)	Ce1^iii^—F9—Ce2	100.8 (2)
Ce1—F3—Cs1^i^	124.6 (3)	Ce1^iii^—F9—Cs1	109.0 (2)
Ce1—F3—Cs2^xiii^	109.0 (2)	Ce1^iii^—F9—F2	115.2 (3)
Ce1—F3—Cs2^i^	127.9 (2)	Ce1^iii^—F9—F3^iii^	43.46 (17)
Ce1—F3—F3^x^	161.8 (3)	Ce1^iii^—F9—F5	50.11 (18)
Ce1—F3—F4	54.6 (2)	Ce1^iii^—F9—F6	108.1 (2)
Ce1—F3—F5	51.44 (19)	Ce1^iii^—F9—F6^iii^	47.52 (16)
Ce1—F3—F5^i^	47.28 (16)	Ce1^iii^—F9—F7	154.3 (2)
Ce1—F3—F6	55.92 (18)	Ce1^iii^—F9—F7^iii^	49.44 (18)
Ce1—F3—F9^i^	68.3 (2)	Ce1^iii^—F9—F8	105.5 (2)
Cs1^xiii^—F3—Cs1^i^	122.4 (2)	Ce1^iii^—F9—F10^i^	141.3 (2)
Cs1^xiii^—F3—Cs2^xiii^	72.60 (14)	Ce2—F9—Cs1	117.2 (2)
Cs1^xiii^—F3—Cs2^i^	81.08 (15)	Ce2—F9—F2	49.88 (18)
Cs1^xiii^—F3—F3^x^	61.33 (18)	Ce2—F9—F3^iii^	129.8 (3)
Cs1^xiii^—F3—F4	77.1 (2)	Ce2—F9—F5	117.3 (3)
Cs1^xiii^—F3—F5	61.03 (19)	Ce2—F9—F6	59.41 (18)
Cs1^xiii^—F3—F5^i^	132.2 (2)	Ce2—F9—F6^iii^	73.93 (19)
Cs1^xiii^—F3—F6	114.8 (3)	Ce2—F9—F7	91.3 (2)
Cs1^xiii^—F3—F9^i^	176.8 (2)	Ce2—F9—F7^iii^	56.16 (18)
Cs1^i^—F3—Cs2^xiii^	74.44 (13)	Ce2—F9—F8	150.9 (3)
Cs1^i^—F3—Cs2^i^	78.80 (16)	Ce2—F9—F10^i^	42.01 (14)
Cs1^i^—F3—F3^x^	61.10 (18)	Cs1—F9—F2	67.43 (18)
Cs1^i^—F3—F4	149.1 (3)	Cs1—F9—F3^iii^	66.80 (18)
Cs1^i^—F3—F5	151.4 (2)	Cs1—F9—F5	124.4 (3)
Cs1^i^—F3—F5^i^	99.7 (2)	Cs1—F9—F6	142.6 (3)
Cs1^i^—F3—F6	85.8 (2)	Cs1—F9—F6^iii^	87.5 (2)
Cs1^i^—F3—F9^i^	58.10 (17)	Cs1—F9—F7	84.64 (19)
Cs2^xiii^—F3—Cs2^i^	122.6 (2)	Cs1—F9—F7^iii^	145.4 (3)
Cs2^xiii^—F3—F3^x^	53.91 (14)	Cs1—F9—F8	65.10 (17)
Cs2^xiii^—F3—F4	136.5 (3)	Cs1—F9—F10^i^	100.6 (2)
Cs2^xiii^—F3—F5	80.86 (19)	F2—F9—F3^iii^	106.4 (3)
Cs2^xiii^—F3—F5^i^	147.0 (2)	F2—F9—F5	161.8 (3)
Cs2^xiii^—F3—F6	59.80 (17)	F2—F9—F6	100.7 (3)
Cs2^xiii^—F3—F9^i^	104.9 (2)	F2—F9—F6^iii^	67.9 (2)
Cs2^i^—F3—F3^x^	68.73 (17)	F2—F9—F7	90.0 (3)
Cs2^i^—F3—F4	81.45 (19)	F2—F9—F7^iii^	95.7 (3)
Cs2^i^—F3—F5	127.7 (2)	F2—F9—F8	124.9 (3)
Cs2^i^—F3—F5^i^	86.59 (18)	F2—F9—F10^i^	54.8 (2)
Cs2^i^—F3—F6	162.4 (3)	F3^iii^—F9—F5	70.9 (2)
Cs2^i^—F3—F9^i^	102.1 (2)	F3^iii^—F9—F6	147.4 (3)
F3^x^—F3—F4	131.3 (3)	F3^iii^—F9—F6^iii^	55.9 (2)
F3^x^—F3—F5	114.2 (3)	F3^iii^—F9—F7	137.0 (3)
F3^x^—F3—F5^i^	150.6 (3)	F3^iii^—F9—F7^iii^	91.5 (3)
F3^x^—F3—F6	111.0 (2)	F3^iii^—F9—F8	78.9 (2)
F3^x^—F3—F9^i^	119.1 (3)	F3^iii^—F9—F10^i^	161.2 (2)
F4—F3—F5	57.1 (2)	F5—F9—F6	77.7 (2)
F4—F3—F5^i^	55.4 (2)	F5—F9—F6^iii^	97.4 (3)
F4—F3—F6	108.7 (3)	F5—F9—F7	104.2 (3)
F4—F3—F9^i^	103.9 (3)	F5—F9—F7^iii^	66.7 (2)
F5—F3—F5^i^	93.2 (2)	F5—F9—F8	72.9 (2)
F5—F3—F6	69.3 (2)	F5—F9—F10^i^	127.3 (3)
F5—F3—F9^i^	116.8 (3)	F6—F9—F6^iii^	122.1 (3)
F5^i^—F3—F6	87.7 (2)	F6—F9—F7	59.2 (2)
F5^i^—F3—F9^i^	49.21 (18)	F6—F9—F7^iii^	67.8 (2)
F6—F3—F9^i^	62.0 (2)	F6—F9—F8	100.1 (3)
Ce1—F4—Ce2^viii^	173.3 (4)	F6—F9—F10^i^	49.8 (2)
Ce1—F4—F1^viii^	125.2 (4)	F6^iii^—F9—F7	157.8 (3)
Ce1—F4—F2^viii^	138.2 (4)	F6^iii^—F9—F7^iii^	58.0 (2)
Ce1—F4—F3	48.98 (18)	F6^iii^—F9—F8	134.0 (3)
Ce1—F4—F5	53.23 (16)	F6^iii^—F9—F10^i^	111.7 (2)
Ce1—F4—F5^i^	53.36 (16)	F7—F9—F7^iii^	126.9 (3)
Ce1—F4—F8	48.67 (18)	F7—F9—F8	59.6 (2)
Ce1—F4—F10^xi^	127.4 (3)	F7—F9—F10^i^	50.06 (18)
Ce1—F4—F10^viii^	126.2 (2)	F7^iii^—F9—F8	139.4 (3)
Ce2^viii^—F4—F1^viii^	48.04 (19)	F7^iii^—F9—F10^i^	92.3 (2)
Ce2^viii^—F4—F2^viii^	48.52 (18)	F8—F9—F10^i^	109.4 (3)
Ce2^viii^—F4—F3	137.6 (4)	Ce2—F10—Ce2^iii^	138.6 (3)
Ce2^viii^—F4—F5	128.2 (3)	Ce2—F10—Cs2^ii^	106.3 (2)
Ce2^viii^—F4—F5^i^	124.8 (2)	Ce2—F10—F1	47.22 (17)
Ce2^viii^—F4—F8	124.7 (4)	Ce2—F10—F1^iii^	118.6 (3)
Ce2^viii^—F4—F10^xi^	53.56 (16)	Ce2—F10—F2	44.21 (16)
Ce2^viii^—F4—F10^viii^	52.57 (15)	Ce2—F10—F2^iii^	142.4 (3)
F1^viii^—F4—F2^viii^	96.5 (2)	Ce2—F10—F4^xv^	157.3 (3)
F1^viii^—F4—F3	168.0 (3)	Ce2—F10—F4^ii^	55.57 (16)
F1^viii^—F4—F5	123.7 (3)	Ce2—F10—F5^ii^	105.4 (2)
F1^viii^—F4—F5^i^	98.5 (3)	Ce2—F10—F6^iii^	80.6 (2)
F1^viii^—F4—F8	77.4 (3)	Ce2—F10—F7^iii^	55.37 (17)
F1^viii^—F4—F10^xi^	70.8 (2)	Ce2—F10—F9^iii^	106.3 (2)
F1^viii^—F4—F10^viii^	63.1 (3)	Ce2^iii^—F10—Cs2^ii^	106.1 (2)
F2^viii^—F4—F3	90.3 (3)	Ce2^iii^—F10—F1	147.4 (3)
F2^viii^—F4—F5	104.6 (3)	Ce2^iii^—F10—F1^iii^	42.08 (17)
F2^viii^—F4—F5^i^	128.9 (3)	Ce2^iii^—F10—F2	113.0 (3)
F2^viii^—F4—F8	167.5 (3)	Ce2^iii^—F10—F2^iii^	47.22 (18)
F2^viii^—F4—F10^xi^	63.5 (2)	Ce2^iii^—F10—F4^xv^	55.32 (16)
F2^viii^—F4—F10^viii^	68.1 (2)	Ce2^iii^—F10—F4^ii^	152.2 (3)
F3—F4—F5	63.3 (2)	Ce2^iii^—F10—F5^ii^	104.9 (2)
F3—F4—F5^i^	69.6 (2)	Ce2^iii^—F10—F6^iii^	58.2 (2)
F3—F4—F8	97.6 (2)	Ce2^iii^—F10—F7^iii^	96.2 (2)
F3—F4—F10^xi^	103.9 (3)	Ce2^iii^—F10—F9^iii^	42.60 (14)
F3—F4—F10^viii^	128.8 (3)	Cs2^ii^—F10—F1	59.42 (18)
F5—F4—F5^i^	106.6 (2)	Cs2^ii^—F10—F1^iii^	134.3 (2)
F5—F4—F8	70.8 (2)	Cs2^ii^—F10—F2	140.5 (3)
F5—F4—F10^xi^	163.6 (4)	Cs2^ii^—F10—F2^iii^	60.40 (18)
F5—F4—F10^viii^	77.6 (2)	Cs2^ii^—F10—F4^xv^	79.2 (2)
F5^i^—F4—F8	63.3 (2)	Cs2^ii^—F10—F4^ii^	85.3 (2)
F5^i^—F4—F10^xi^	76.2 (2)	Cs2^ii^—F10—F5^ii^	80.24 (16)
F5^i^—F4—F10^viii^	158.1 (4)	Cs2^ii^—F10—F6^iii^	146.5 (3)
F8—F4—F10^xi^	123.2 (3)	Cs2^ii^—F10—F7^iii^	96.0 (2)
F8—F4—F10^viii^	99.3 (3)	Cs2^ii^—F10—F9^iii^	100.21 (19)
F10^xi^—F4—F10^viii^	106.1 (2)	F1—F10—F1^iii^	165.7 (3)
Ce1—F5—Ce1^iii^	135.5 (3)	F1—F10—F2	88.8 (2)
Ce1—F5—Cs1^xiii^	111.0 (2)	F1—F10—F2^iii^	106.5 (3)
Ce1—F5—F3	47.69 (18)	F1—F10—F4^xv^	135.6 (3)
Ce1—F5—F3^iii^	106.9 (2)	F1—F10—F4^ii^	60.2 (2)
Ce1—F5—F4	54.91 (17)	F1—F10—F5^ii^	101.2 (2)
Ce1—F5—F4^iii^	146.6 (3)	F1—F10—F6^iii^	116.1 (2)
Ce1—F5—F6	47.24 (15)	F1—F10—F7^iii^	59.9 (2)
Ce1—F5—F7^iii^	101.7 (2)	F1—F10—F9^iii^	107.6 (2)
Ce1—F5—F8	42.39 (17)	F1^iii^—F10—F2	77.1 (2)
Ce1—F5—F8^iii^	155.2 (3)	F1^iii^—F10—F2^iii^	86.6 (2)
Ce1—F5—F9	62.5 (2)	F1^iii^—F10—F4^xv^	56.2 (2)
Ce1—F5—F10^viii^	103.2 (2)	F1^iii^—F10—F4^ii^	112.4 (3)
Ce1^iii^—F5—Cs1^xiii^	110.3 (2)	F1^iii^—F10—F5^ii^	80.1 (2)
Ce1^iii^—F5—F3	150.7 (3)	F1^iii^—F10—F6^iii^	55.9 (2)
Ce1^iii^—F5—F3^iii^	42.70 (15)	F1^iii^—F10—F7^iii^	115.7 (3)
Ce1^iii^—F5—F4	149.7 (3)	F1^iii^—F10—F9^iii^	76.1 (2)
Ce1^iii^—F5—F4^iii^	53.13 (16)	F2—F10—F2^iii^	159.0 (3)
Ce1^iii^—F5—F6	105.16 (19)	F2—F10—F4^xv^	118.6 (3)
Ce1^iii^—F5—F7^iii^	52.32 (16)	F2—F10—F4^ii^	57.1 (2)
Ce1^iii^—F5—F8	110.0 (2)	F2—F10—F5^ii^	84.2 (2)
Ce1^iii^—F5—F8^iii^	46.43 (17)	F2—F10—F6^iii^	66.1 (2)
Ce1^iii^—F5—F9	73.1 (2)	F2—F10—F7^iii^	85.8 (2)
Ce1^iii^—F5—F10^viii^	102.6 (2)	F2—F10—F9^iii^	112.3 (2)
Cs1^xiii^—F5—F3	65.45 (18)	F2^iii^—F10—F4^xv^	59.6 (2)
Cs1^xiii^—F5—F3^iii^	139.2 (3)	F2^iii^—F10—F4^ii^	143.2 (3)
Cs1^xiii^—F5—F4	80.3 (2)	F2^iii^—F10—F5^ii^	106.2 (2)
Cs1^xiii^—F5—F4^iii^	84.7 (2)	F2^iii^—F10—F6^iii^	93.8 (3)
Cs1^xiii^—F5—F6	106.0 (2)	F2^iii^—F10—F7^iii^	89.5 (3)
Cs1^xiii^—F5—F7^iii^	103.7 (2)	F2^iii^—F10—F9^iii^	49.86 (18)
Cs1^xiii^—F5—F8	135.8 (2)	F4^xv^—F10—F4^ii^	104.0 (2)
Cs1^xiii^—F5—F8^iii^	64.89 (17)	F4^xv^—F10—F5^ii^	53.02 (19)
Cs1^xiii^—F5—F9	155.3 (3)	F4^xv^—F10—F6^iii^	107.2 (3)
Cs1^xiii^—F5—F10^viii^	75.28 (16)	F4^xv^—F10—F7^iii^	147.1 (3)
F3—F5—F3^iii^	154.5 (3)	F4^xv^—F10—F9^iii^	94.1 (2)
F3—F5—F4	59.6 (2)	F4^ii^—F10—F5^ii^	51.12 (17)
F3—F5—F4^iii^	146.3 (3)	F4^ii^—F10—F6^iii^	122.9 (3)
F3—F5—F6	53.13 (19)	F4^ii^—F10—F7^iii^	108.0 (2)
F3—F5—F7^iii^	99.3 (2)	F4^ii^—F10—F9^iii^	161.8 (2)
F3—F5—F8	87.2 (2)	F5^ii^—F10—F6^iii^	130.4 (3)
F3—F5—F8^iii^	116.3 (3)	F5^ii^—F10—F7^iii^	158.8 (3)
F3—F5—F9	99.2 (3)	F5^ii^—F10—F9^iii^	146.7 (2)
F3—F5—F10^viii^	104.0 (2)	F6^iii^—F10—F7^iii^	60.4 (2)
F3^iii^—F5—F4	111.0 (3)	F6^iii^—F10—F9^iii^	47.38 (19)
F3^iii^—F5—F4^iii^	55.0 (2)	F7^iii^—F10—F9^iii^	54.4 (2)

Symmetry codes: (i) *x*, −*y*+3/2, *z*−1/2; (ii) *x*+1, −*y*+3/2, *z*+1/2; (iii) *x*, −*y*+3/2, *z*+1/2; (iv) −*x*+2, *y*−1/2, −*z*+1/2; (v) −*x*+2, −*y*+1, −*z*+1; (vi) −*x*+1, *y*−1/2, −*z*+1/2; (vii) −*x*+1, −*y*+1, −*z*; (viii) *x*−1, −*y*+3/2, *z*−1/2; (ix) *x*−1, *y*, *z*; (x) −*x*+1, −*y*+2, −*z*; (xi) *x*−1, *y*, *z*−1; (xii) −*x*+2, *y*+1/2, −*z*+1/2; (xiii) −*x*+1, *y*+1/2, −*z*+1/2; (xiv) *x*+1, *y*, *z*; (xv) *x*+1, *y*, *z*+1.
